# Hormone replacement therapy perspectives

**DOI:** 10.3389/fgwh.2024.1397123

**Published:** 2024-05-20

**Authors:** Dohn Kissinger

**Affiliations:** Independent Researcher, El Dorado Hills, California, CA, United States

**Keywords:** HRT, MHT, WHI, Prempro, MPA, bioidentical, progestin, progesterone

## Abstract

Hormone replacement therapy (HRT), also known as menopausal hormone therapy (MHT), was looked upon as a fountain of youth that kept women young and reduced cardiovascular disease. This led to a large-scale study called the Women's Health Initiative (WHI) that was conducted to show the cardiovascular benefits of HRT. This study was suspended early because of adverse side effects. The USFDA responded by slapping a “black box” warning on all HRT products. USFDA-approved bioidentical HRT formulations are safe and effective. We propose that these formulations have the “black box” warning removed so that doctors feel more confident in prescribing these products for symptoms of menopause and chronic conditions such as cardiovascular health. We propose eliminating the sale of products containing medroxyprogesterone acetate (MPA) because of the increased risk of heart attacks and breast cancers associated with this medication.

## Introduction

There is widespread confusion about hormone replacement therapy (HRT). This confusion has occurred primarily because of misinterpretation of results from the Women's Health Initiative (WHI) study conducted in the 1991-early 2000 timeframe. The WHI study was suspended because of adverse side effects on the study participants. Many commentators declared that the concept of HRT was “dead”.

## Discussion

Analysis of the results from the Women's Health Initiative (WHI) study (1) revealed that participants who took Prempro, a combination of Premarin (conjugated equine estrogen) plus medroxyprogesterone acetate (MPA) had a higher incidence of side effects, including breast cancer and heart attacks, than control group participants who were assigned a placebo. On the other hand, hysterectomized participants who took only Premarin had a lower incidence of side effects than control group participants who were assigned a placebo. It can therefore be concluded that MPA is the culprit in increasing the likelihood of side effects of HRT, not the estrogen. This article concluded that “Our analysis suggests that failure to differentiate among populations of women and preparations of HT has cost thousands of lives”.

The USFDA generalized the WHI results to assume that any HRT products were dangerous to women's health. The USFDA requires a “black box” warning on all USFDA-approved HRT products that includes the statement: “A study of women taking an estrogen with a progestin showed a raised risk of heart attack, stroke, blood clot, breast cancer, and dementia”.

Because MPA is a harmful synthetic progestin does not mean that all synthetic progestins are harmful. Another study investigated the effect of HRT containing the synthetic progestin norethisterone acetate on recently postmenopausal women in Denmark (2). The conclusion of the study was “Our findings suggest that initiation of hormone replacement therapy in women early after menopause significantly reduces the risk of the combined endpoint of mortality, myocardial infarction, or heart failure. Importantly, early initiation and prolonged hormone replacement therapy did not result in an increased risk of breast cancer or stroke”.

It seems clear from these two studies that the HRT therapies that do not include MPA result in improvement in women's cardiovascular health. Unfortunately, the USFDA prohibits statements that support the use of any HRT formulations to improve cardiovascular health, because they assume that the risks are high, based on the results of the WHI study that included the use of MPA.

Many HRT therapies have been approved by the USFDA that consist of bioidentical HRT products. The term “bioidentical” means the hormones in the product are chemically identical to those that the body produces. Estradiol is a bioidentical estrogen that is available in pills, patches, sprays, creams, gels, and vaginal tablets. Transdermal estradiol applications seem to be more desirable than estrogen pills because the estradiol does not go through the liver. If the uterus is intact, progestogens are added to the estrogen therapy to reduce the risk of cancer of the uterus. Bioidentical progestogen products include micronized progesterone, such as Prometrium. Recently, the USFDA has approved Bijuva, a combination pill that includes both bioidentical estradiol and bioidentical progesterone.

Campagnoli et al. (3) reviewed the influence of progestins and progesterone on the risk of breast cancer (BC) to provide suggestions for the prescription of HRT in climacteric women. The conclusion of this review was “many of the progestins used have several non-progesterone like actions that potentiate the proliferative effect of estrogens on breast tissue and estrogensensitive cancer cells. We therefore suggest that when HRT is indicated, preparations containing progesterone and not a synthetic progestin should be used, according to a sequential or cyclic-combined regimen. In this way the risk of endometrial cancer is minimized without increasing the risk of BC”.

Asi et al. (4) conducted a systematic review and meta-analysis to synthesize the existing evidence about the effect of progesterone in comparison to synthetic progestins, each in combination with estrogens, on the risk of breast cancer and cardiovascular events. The conclusions of this review and analysis were “Observational studies suggest that in menopausal women taking estrogen, progesterone use may be associated with lower breast cancer risk compared to synthetic progestin”.

Another study (5) concluded that “The evidence is clear that progesterone does not cause breast cancer. Indeed, progesterone is protective and preventative of breast cancer”.

How long can women use HRT? It used to be the rule that women should use HRT for the shortest time possible. However, it has been shown that the benefits of estrogen decrease after discontinuing HRT. Therefore, The Menopause Society, formerly the North American Menopause Society (NAMS), states in their 2022 hormone therapy position statement (6), “There is no general rule for stopping systemic hormone therapy in a woman aged 65 years. The Beers criteria from the American Geriatrics Society has warnings against the use of hormone therapy in women aged older than 65 years. However, the recommendation to routinely discontinue systemic hormone therapy in women aged 65 years and older is neither cited or supported by evidence nor is it recommended by the American College of Obstetricians and Gynecologists or The North American Menopause Society”.

What is HRT not recommended for? The NAMS 2022 hormone therapy position statement does not recommend compounded HRT products, because “Compounded bioidentical hormone therapy presents safety concerns, such as minimal government regulation and monitoring, overdosing and underdosing, presence of impurities and lack of sterility, lack of scientific efficacy and safety data, and lack of a label outlining risks”.

The US Preventive Services Task Force (USPSTF) does not recommend that HRT be used to improve chronic conditions, such as cardiac health. They state (7), “The USPSTF concludes with moderate certainty that the use of combined estrogen and progestin for the primary prevention of chronic conditions in postmenopausal persons with an intact uterus has no net benefit. The USPSTF concludes with moderate certainty that the use of estrogen alone for the primary prevention of chronic conditions in postmenopausal persons who have had a hysterectomy has no net benefit”. The USPSTF statements regarding HRT therapies for improving chronic conditions are based on the perceived unfavorable benefit-to-risk ratio of HRT using synthetic progestins. Since USFDA-approved bioidentical HRT medications are low risk, the benefit-to-risk ratio of these medications concerning heart health will be favorable, so they are a viable approach to improving women's cardiovascular health and other chronic conditions.

## Conclusions and recommendations

1. The “black box” warning should be eliminated for all bioidentical USFDA-approved HRT products. This would enable all women to receive the benefit of HRT therapy without the concern for adverse side effects.
2. USFDA-approved bioidentical HRT medications are both safe and effective. Medical providers should be encouraged to inform menopausal women about these medications to relieve symptoms of menopause and improve heart health.
3. HRT products containing MPA result in women risking higher rates of heart attacks and breast cancers using these products. The sale of these products should be banned.
4. Compounded HRT products are higher risk than USFDA-approved HRT products, and should be avoided when possible.
